# Novel Approaches to Treatment of Advanced Melanoma: A Review on Targeted Therapy and Immunotherapy

**DOI:** 10.1155/2015/851387

**Published:** 2015-06-10

**Authors:** Anna Niezgoda, Piotr Niezgoda, Rafał Czajkowski

**Affiliations:** ^1^Department of Dermatology, Sexually Transmitted Diseases and Immunodermatology, Faculty of Medicine, Nicolaus Copernicus University in Toruń, 9 Sklodowskiej-Curie Street, 85-094 Bydgoszcz, Poland; ^2^Department of Cardiology and Internal Medicine, Faculty of Medicine, Nicolaus Copernicus University in Toruń, 9 Sklodowskiej-Curie Street, 85-094 Bydgoszcz, Poland

## Abstract

The incidence of malignant melanoma is increasing. The majority of patients are diagnosed in early stages when the disease is highly curable. However, the more advanced or metastatic cases have always been a challenge for clinicians. The poor prognosis for patients with melanoma is now changing as numerous of promising approaches have appeared recently. The discovery of aberrations of pathways responsible for intracellular signal transduction allowed us to introduce agents specifically targeting the mutated cascades. Numerous clinical studies have been conducted to improve effectiveness of melanoma treatment. From 2011 until now, the U.S. FDA has approved seven novel agents, such as BRAF-inhibitors (vemurafenib 2011, dabrafenib 2013), MEK-inhibitors (trametinib 2013), anti-PD1 antibodies (nivolumab 2014, pembrolizumab 2014), anti-CTLA-4 antibody (ipilimumab 2011), or peginterferon-alfa-2b (2011) intended to be used in most advanced cases of melanoma. Nevertheless, clinicians continue working on new possible methods of treatment as resistance to the novel drugs is a commonly observed problem. This paper is based on latest data published until the end of January 2015.

## 1. Introduction

**Figure 1:**
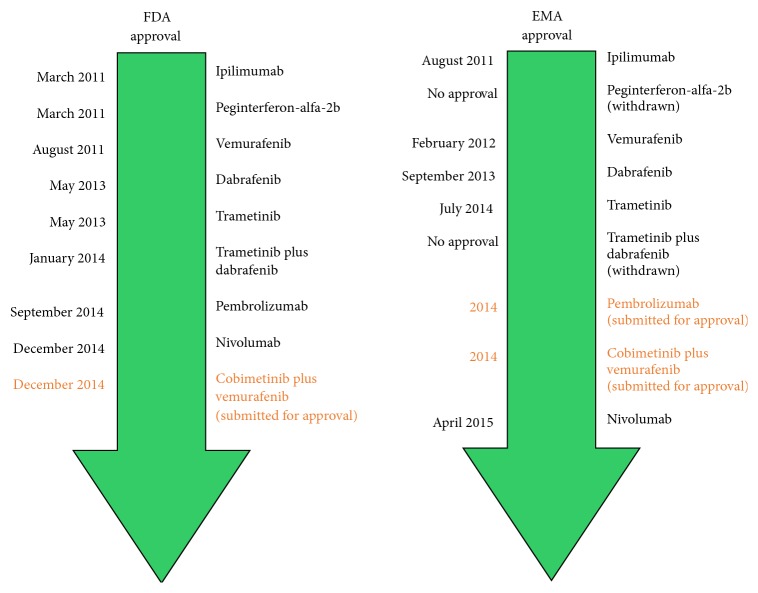
The time axis presenting dates of FDA (US Food and Drug Administration) and EMA (European Medicines Agency) approval of novel agents for advanced melanoma treatment.

Over the past decades the incidence of malignant melanoma tends to be increasing [1]. According to the data provided by the WHO about 132,000 melanoma skin cancers are being diagnosed each year globally [2]. Melanoma has been reported as the fifth and seventh most common cancer type in the United States in men and women, respectively, excluding basal cell and squamous-cell skin cancer as well as in situ carcinoma except urinary bladder cancer [3]. As it is estimated by the National Cancer Institute about 73,870 new cases of melanoma (42,670 in men and 31,200 in women) will be diagnosed in 2015 in the US and the number of deaths from the disease will reach 9940 [3]. The incidence of melanoma additionally varies by ethnic group. It accounts for 1 (per 100,000) in black people, 4 in Hispanics, and 25 in non-Hispanic whites annually [3]. Following the US NCI as of January 1, 2014, the number of melanoma survivors is estimated at about 528,860 women and 516,570 men. Almost two-thirds of all melanoma survivors in the US are younger than 70 years old and moreover about 215,820 of them are younger than 50 years old [1]. Patients are diagnosed with melanoma at the median age of 64 years for men and 57 years for women [4]. As of January 1, 2024, the numbers are supposed to reach 696,280 women and 698,040 men [1]. The vast majority of melanomas are diagnosed in the early stage; thus, they are in most cases curable. The more advanced cases are still a great challenge to face though. The 5-year survival for all stages of melanoma is in average 91%. Patients with localized melanoma have the 5-year survival rate of about 98%, but the rate radically declines in regional and distant stage disease to reach 63% and 16%, respectively [3]. The treatment of melanoma varies depending on the stage of the disease. According to the NCI surgical excision is a method of choice for stage 0 melanoma, excision and lymph node management for stages I, II, and resectable III melanoma, and immunotherapy, chemotherapy, targeted therapy, or palliative local therapy for unresectable stage III, stage IV, and recurrent melanoma [5]. Last few years brought a major breakthrough related to the treatment of advanced melanoma. The most important milestones were the approval of immune checkpoint inhibitors such as nivolumab, ipilimumab, and pembrolizumab, as well as the introduction of targeted therapy, which consists of BRAF protein inhibitors such as vemurafenib and dabrafenib or MEK inhibitors represented by trametinib (Figure 1). Moreover, there are many ongoing clinical trials testing the efficacy and safety of the new molecules destined to treat the advanced cases of melanoma.

## 2. Molecular Basics of Pathogenesis of Melanoma

**Figure 2:**
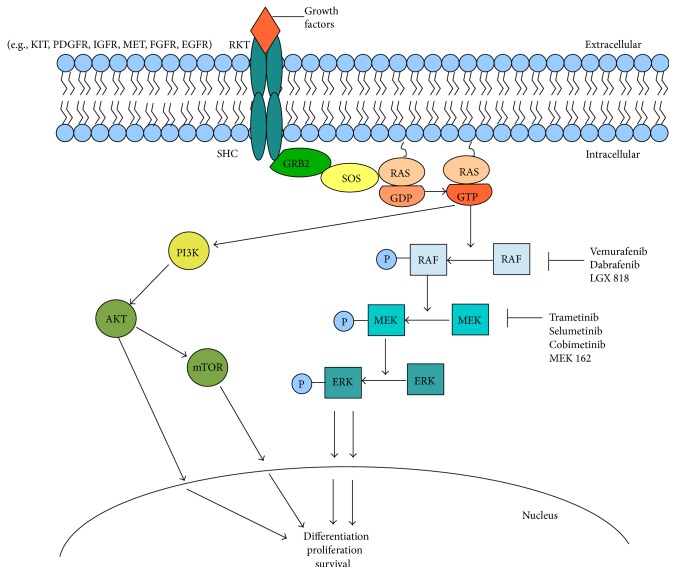
Mitogen-activated protein kinase (MAPK) signalling pathway. MAPK pathway is responsible for differentiation, proliferation, and survival of the cells. Mutations on particular stages of the pathway lead to uncontrolled enhancements of these processes.

Many years of clinical trials of the processes of transformation of the melanocytes into invasive melanoma cells led to the discovery of numerous mechanisms responsible for growth and spreading of the cancer. Melanoma is heterogeneous; its pathogenesis partly depends on DNA mutations which lead to the activation of oncogenes or to the inactivation of the suppressor genes as well as the amplification of parts or whole chromosomes. The aberrations mentioned above lead in turn to karyotypic profiles which differ in various subtypes of melanoma. Several intracellular signaling pathways have been studied so far, the best known of which is definitely the mitogen activated protein kinase (MAPK) pathway or RAS-RAF-MEK-ERK pathway (Figure 2) [6]. The intracellular MAPK pathway can be activated by various extracellular impulses. Growth factors such as EGF (epidermal growth factor), IGF (insulin-like growth factor), or TGF (transforming growth factor) induce signal transduction by binding to the transmembrane receptors located on the surface of a cell. This in turn leads to the activation of the RAS protein which transducts the signal to the group of serine-threonine kinases RAF, including ARAF, BRAF, and CRAF. Each of those three kinases may activate the next stage of the pathway, MEK1 or MEK2 kinases. The following stage of the pathway is kinases ERK1 and ERK2 that either phosphorylate cytoplasmic proteins or migrate into the cell nucleus influencing the transcription factors regulating proliferation, differentiation, and genes connected with these processes [7–9].

It has been estimated that about 50% of melanomas contain MAPK pathway activating mutations, which results in considering it the most important therapeutic target [10]. Over 75 somatic mutations in the BRAF gene have been described so far [11]. The most commonly observed mutation in melanomas with no chronic sun damage is V600E resulting in substitution of glutamic acid for valine, which ranges in frequency from 30% to 72% [12, 13]. The second most common BRAF mutation is BRAF V600K substituting lysine for valine, which represents 5-6% [14]. There are also mutations in another signalling pathway PI3K/AKT/mTOR and the suppressor gene PTEN, but they are observed at lower frequencies [15].

### 2.1. The Role of Immune Regulation

**Figure 3:**
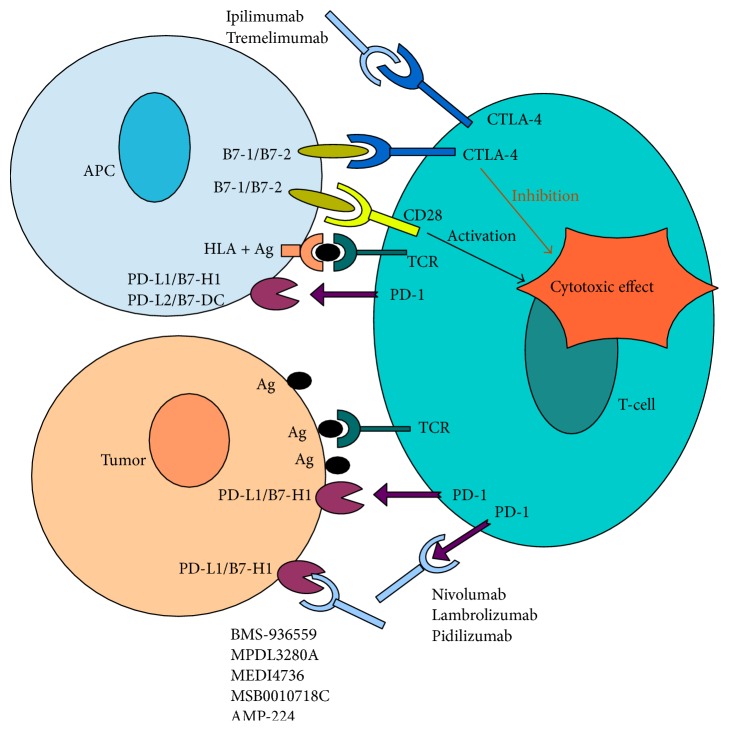
T-cell activation is a consequence of two simultaneous processes, the expression of protein B7 and antigen presentation by antigen-presenting cells (APCs) to T-cell receptor. The inhibition of activated T-cells occurs as a result of the expression of CTLA-4 and PD-1 receptor on T-cells' surface.

Another link in understanding the pathophysiology of melanoma is the immune regulation of T-cells. T-cells are covered with various receptors which are the target of antigen-presenting cells (APCs). The antigen presentation in turn leads to activation or inactivation of the T-cell. The activation of a T-cell occurs by two concurrent mechanisms which consist of antigen presentation by APCs to the T-cell receptor (TCR) and the expression (by APCs as well) of protein B7 which interacts with the T-cell CD28 receptor. If these two processes occur simultaneously, they trigger the stimulatory impulses to the nucleus, which leads to the activation of the T-cell. Nevertheless, there are numerous pathways of inactivation of once activated T-cells. The phenomena mentioned above consist of (1) the expression of the CTLA-4 receptor on the surface of a T-cell, which after binding to the protein B7 on APCs transducts the inhibitory signal to the nucleus and (2) the expression of the PD-1 receptor on the T-cell surface which may lead to the inactivation of the T-cell after binding to the PD-L1 (programmed cell death 1 ligand) on tumor tissue. The inhibition or checkpoint blockade of CTLA-4 or PD-1 (or PD-L1) may thus be used as the target of the antitumor treatment (Figure 3) [36–38].

## 3. Targeted Therapy

### 3.1. BRAF Inhibitors

#### 3.1.1. Vemurafenib

The discovery of the mutations concerning BRAF allowed us to introduce the inhibitors of the mutant BRAF kinases. The agent that should be considered a significant breakthrough is definitely vemurafenib, a highly specific inhibitor of the BRAF kinase that harbours the mutation V600. Several attempts to inhibit the BRAF kinases had been performed before the discovery of vemurafenib, especially using sorafenib, the nonspecific BRAF inhibitors, but they all turned out to be a failure eventually due to the insufficient clinical activity or the hardly acceptable adverse effects of the drug. The clinical trials of vemurafenib began in 2008 but shortly after, in 2011, it was approved by the FDA to treat late-stage or unresectable melanoma [16]. Before vemurafenib was introduced, dacarbazine was the drug of choice for metastatic melanoma, in spite of its low clinical activity and poor response rates ranging from 11 to 25% and median survival time of 4, 5 to 6 months [17]. The study that contributed most to the development of vemurafenib as the treatment for patients with metastatic melanoma was BRIM. In phase I (BRIM-1) patients with advanced tumors, the majority of whom had metastatic melanoma with BRAF V600E mutation (89%), underwent treatment with vemurafenib. The trial consisted of two stages where patients were grouped into the dose-escalation cohort and the dose-expansion cohort. Being given the dose up to 720 mg twice daily, the patients did not develop dose-limiting toxicities. However, adverse effects such as arthralgia, nausea, fatigue, rash, and photosensitivity were observed quite commonly. Among the patients in the dose-escalation group about 69% (11 out of 16 who harbored V600E mutation) experienced a response, whereas in the dose-expansion group 26 out of 32 patients with melanoma with V600E mutation met the criteria for ORR (overall response rate) on the dose of 960 mg twice daily. The PFS (progression-free survival) in dose-escalation cohort reached more than 7 months while the median survival was about 13.8 months [16]. The results mentioned above suggested 960 mg twice daily as an appropriate dose for phase II trial.

Phase II trial (BRIM-2) enrolled 132 patients with BRAF V600 mutation previously treated for metastatic melanoma. All the patients were administered vemurafenib 960 mg twice daily. The primary endpoint of the study was to measure ORR as determined by RECIST v1.1 and the overall survival was the secondary endpoint. The confirmed ORR was 53% which included 6% of patients with complete response (CR) and 47% with partial response (PR). The median PFS was 6, 8 months while the primary progression was observed in only 14% of patients [17]. About 29% of patients had stable disease. The group of 33% of patients presented inferior response. After the precise analysis of the subgroups of patients it turned out that the patients from the group with the inferior response had an elevated baseline level of lactate dehydrogenase about 1, 5 times greater than normal. The median OS (overall survival) was 15, 9 months [16]. To sum up, phase II showed that vemurafenib induced clinical response in more than 50% of patients with previously treated metastatic melanoma with BRAF V600 mutation and the follow-up revealed the overall survival of approximately 16 months.

The crucial trial for vemurafenib to be approved for treatment was BRIM-3, a phase III study comparing vemurafenib to dacarbazine in unresectable, previously untreated melanoma in stage IIIC or IV with BRAF V600 mutation [18]. The primary endpoints of the study included OS and PFS. The secondary endpoints included the proportion of patients with a confirmed response (either complete or partial), time to response, duration of response, tolerability and safety of vemurafenib, time to treatment failure, and the pharmacokinetic profile of vemurafenib. Overall, 2107 patients older than 18 years were screened and 675 of them enrolled in the study between January 4, 2010, and December 16, 2010. They were randomized into two groups, 337 patients receiving vemurafenib 960 mg twice daily and 338 patients receiving dacarbazine 1000 mg/m^2^ every three weeks. A total number of 399 patients (59%) had died before the data cutoff (February 1, 2012). The mentioned treatment continued until progression of the disease or unacceptable toxicity. During the trial the tumor assessments were conducted at baseline, week 6, week 12, and subsequently every 9 weeks. The final analysis was planned after 196 deaths and an interim analysis after 98 deaths. The overall survival at 6 months was 84% (95% CI, 78–89) in the vemurafenib group and 64% (95% CI, 56–73) in the dacarbazine group. Vemurafenib was also associated with a 63% reduction of the risk of death and 73% reduction of the risk of either death or disease progression as compared with dacarbazine. After review of an interim analysis conducted by an independent review committee crossover from dacarbazine arm to vemurafenib arm was recommended for patients who progressed while undergoing chemotherapy [19]. The median ORR was 48% with PFS of 5.3 months in vemurafenib group and 5% with PFS of 1.6 months in dacarbazine group. The evaluation of the trial showed the median OS of 13.2 months (95% CI, 12.0–15.0) for vemurafenib arm and 9.6 months (95% CI, 7.9–11.8) for dacarbazine [39]. The most commonly observed adverse effects of the treatment in vemurafenib group were arthralgia, rash, photosensitivity skin reactions, fatigue, nausea, alopecia, pruritus, hyperkeratosis, diarrhea, headache, and vomiting. 61 patients (18%) developed cutaneous squamous-cell carcinoma, keratoacanthoma, or both, which were all treated by simple excision. In dacarbazine group the most common adverse effects included fatigue, nausea, vomiting, and neutropenia. In 129 of 336 patients (38%) in vemurafenib group and in 44 of 282 patients (16%) in dacarbazine group the observed adverse effects forced the drug dose modification or interruption [19].

#### 3.1.2. Dabrafenib

Another BRAF inhibitor used for treatment of melanoma is dabrafenib. In the United States it was approved by the FDA in 2013 as a single-agent treatment for unresectable or metastatic melanoma with BRAF V600E mutation [40, 41]. The trial which contributed significantly to the approval was the BREAK trial. In phase I of the trial 184 patients were enrolled, 156 of whom had melanoma and 28 of whom had other nonmelanoma solid tumors. The treatment continued until disease progression, intolerable toxic events, or withdrawal of consent. The tumor response was assessed with RECIST v1.0. The most common adverse effects included cutaneous squamous-cell carcinoma (20 patients, 11%), fatigue (14, 8%), and pyrexia (11, 6%). Dose reduction was required in 13 patients (7%). There were no deaths or discontinuations because of the adverse effects of the medicament. On the basis of safety and the pharmacokinetic profile of dabrafenib, the dose of 150 mg twice daily was assessed as the recommended dose for phase II. Among the 36 patients with BRAF V600-mutant melanoma being administered the recommended dose, 18 (50%, 32.9–67.1) had confirmed response and the rate for patients with BRAF V600E was 56% (15 out of 27, 56%, 35.3–74.5). (33) The median duration of response was 6.2 months (95% CI, 4.2–7.7). Patients with BRAF V600E and V600K mutations had similar PFS of 5.5 and 5.6 months, respectively [42]. In phase II study patients received dabrafenib 150 mg twice daily until the patient's death, disease progression, or unacceptable adverse effects. The primary endpoint of the study was to measure ORR (either complete response or partial response assessed by an investigator) in patients with BRAF V600E-mutant melanoma. The secondary objectives included ORR in patients with BRAF V600K-mutant melanoma, PFS, OS, duration of response, and characterization of the safety profile of dabrafenib. Disease progression and response evaluations were defined according to RECIST v1.1. In total, 92 patients out of 211 screened were enrolled in the study. All of them had histologically confirmed BRAF V600 mutation, 76 of them had BRAF V600E mutation (83%), and 16 (17%) had BRAF V600K mutation. Most patients had already undergone surgical treatment (98%) and the majority of the patients enrolled (84%) had been given any kind of anticancer therapy, especially chemotherapy. The most commonly used chemotherapeutic agents used in those patients included dacarbazine (46%) or temozolomide (24%). The study showed that the ORR (either partial or complete) was significantly higher in the group of patients harboring BRAF V600E mutation when compared with those with BRAF V600K-mutation (59% (95% CI 48.2–70.3) and 13% (95% CI 0.0–28.7), resp.) [20]. The median PFS was also longer in the BRAF V600E group than in the BRAF V600K group (6.3 months versus 4.5 months) [20]. According to the updated OS analysis the median OS was 13.1 months for BRAF V600E and 12.9 months for BRAF V600K. The 6-month OS was 74% and 73%, respectively, and 1-year OS was 57% and 53%, respectively [20]. As far as the adverse events are concerned the most commonly observed ones include arthralgia (33%), hyperkeratosis (27%), pyrexia (24%), fatigue (22%), headache (21%), and nausea (20%). Serious adverse events were observed in 25 patients (27%) and included basal cell carcinoma (4%), cutaneous squamous-cell carcinoma (9%), anemia (3%), pyrexia (3%), noncardiac chest pain (2%), and vomiting (2%). No cardiac toxicity or valvular abnormalities were described during the evaluation of the trial as it was suggested by the preclinical studies in dogs [20].

Phase III trial (BREAK-3) enrolled patients with stage IV or unresectable stage IIIC melanoma harboring BRAF V600E mutation. Two hundred fifty patients were randomized in a 3 : 1 design into dabrafenib group which consisted of 187 patients receiving dabrafenib 150 mg twice daily and dacarbazine group consisting of 63 patients receiving dacarbazine 1000 mg/m^2^ every 3 weeks. The patients in dacarbazine group were supposed to cross over to dabrafenib in case of disease progression. The primary endpoint was PFS assessed by an investigator, the secondary endpoints included PFS assessed by an independent review committee (IRC), OS and ORR as determined by RECIST v1.1, and PFS after crossover from dacarbazine to dabrafenib. An investigator-assessed progression-free survival was relevantly longer in the dabrafenib group when compared with dacarbazine group (5.1 versus 2.7 months, resp.) and the hazard ratio for progression was 0.30 (95% CI, 0.18–0.51, *p* < 0.0001). PFS assessed by IRC was 6.7 months for dabrafenib and 2.9 months for dacarbazine, and hazard ratio for progression was 0.35 (95% CI, 0.2–0.61). Forty-four percent of patients crossed over to dabrafenib group after disease progression. Confirmed response rates (partial or complete) assessed by IRC were observed in 50% of patients in dabrafenib arm and only 6% in dacarbazine arm. The most common adverse events of the treatment were hyperkeratosis, papillomas, palmar-plantar erythrodysesthesia, pyrexia, fatigue, headache, and arthralgia. Cutaneous squamous-cell carcinoma and cutaneous keratoacanthoma were seen in twelve patients (6%), three patients (2%) developed primary melanoma, and phototoxicity was observed in 3% of the patients [21, 22].

The BREAK-MB trial was designed for patients with V600E or V600K-mutant melanomas and metastases to the brain with or without previous local treatment such as brain surgery, whole-brain radiation, and stereotactic radiosurgery [43]. In the group of patients with V600E mutation with no prior treatment the intracranial response was observed in 39%, with a complete response in 3% of the patients. Median PFS for this group was 16.1 months [43]. In the group of patients with V600E mutation who had undergone local treatment before the study 31% presented partial response and the median PFS for this group was 16.6 weeks. The median OS for both cohorts was 31–33 weeks. The response rates for patients with V600K mutations were lower. The intracranial response could be seen in 7% and 22% for untreated and treated patients, respectively. No complete responses in this group were observed. The median PFS was 8.1 weeks for previously untreated and 15.9 weeks for previously treated patients. The median OS for those cohorts ranged from 16 to 22 weeks [21, 43]. To sum up, the BREAK-MB study suggests that dabrafenib can be considered as a good therapeutic choice for patients with advanced melanoma with brain metastases.

#### 3.1.3. LGX818

Another BRAF inhibitor which is currently under development for BRAF-mutant melanoma is LGX818. The agent causes noticeably longer inhibition of the MAPK pathway when compared to vemurafenib or dabrafenib [44]. The trial regarding LGX818 enrolled 54 patients with various BRAF V600 mutations regardless of the history of treatment with BRAF inhibitors (26 BRAF-inhibitor naive and 28 previously treated with BRAF inhibitor). The recommended phase II dose (RP2D) for LGX818 was 450 mg once daily. The confirmed response to the treatment was observed in 58% of BRAF-naive patients and 11% in previously treated participants. The toxicities observed in patients treated with other BRAF inhibitors, vemurafenib and dabrafenib, such as photosensitivity, liver aminotransferase elevation, or pyrexia, were rare [36, 45, 46]. The trial of LGX818 (COLUMBUS) is currently underway, assessing the efficacy of the combination of LGX818 and MEK inhibitor MEK162 and LGX818 monotherapy versus vemurafenib alone in advanced melanoma harboring V600 mutation [47].

#### 3.1.4. Resistance to Treatment

In spite of the clinical benefit associated with the treatment with BRAF inhibitors, most patients develop resistance to these agents within 6–8 months. The potential mechanisms of resistance include intrinsic resistance or acquired resistance consisting of either ERK-dependent or non-ERK-dependent mechanism. The intrinsic resistance may be caused by several different abnormalities regarding the cell cycle regulation. The amplification in cyclin D1, which can be observed in 15–20% of BRAF-mutant melanomas, is associated with a higher rate of resistance to BRAF inhibitors [48, 49]. Another biomarker that can be used for predicting the probability of resistance to BRAF inhibitors is the status of the suppressor gene, PTEN. As it was reported, PTEN loss is associated with resistance to BRAF inhibitors [50]. On the other hand, tissue expression of PTEN was associated with shorter PFS in patients treated with dabrafenib [51]. Another mechanism which plays the important role in resistance to BRAF inhibitors is the interaction between hepatocyte growth factor (HGF) and its receptor CMET. As it was shown in the trial the addition of HGF or CMET inhibitor leads to reestablishment of sensitivity to BRAF inhibitors [52].

As far as the acquired mechanisms of resistance are concerned, the non-ERK dependent mechanism includes the activation of PI3K/AKT/mTOR pathway by PDGFR*β* (platelet derived growth factor receptor beta) and IGF-1R (insulin-like growth factor 1 receptor). The ERK dependent mechanism includes the potentiation of the signaling through MAPK pathway in nonmutant cells which is observed after blockage of the signal transduction in MAPK pathway in mutant cells that occurs during treatment with BRAF inhibitors such as vemurafenib or dabrafenib. The hyperactivation of MAPK pathway is also seen as a result of upregulation of RTK (receptor tyrosine kinase) and RAF dimer formation [53]. The ERK dependent mechanisms of resistance to BRAF inhibitors also involve the overexpression of the mutant oncoprotein but this particular mechanism can be overcome by increasing the dose of the BRAF inhibitor [54].

Another possible way of overcoming the resistance to BRAF inhibitors seems to be the combination of immunotherapeutic agents and BRAF inhibitors. In 2011 the trial of combination of vemurafenib and ipilimumab was launched so as to measure the efficacy of this combination in advanced melanoma with BRAF V600 mutation. Until now, there are several ongoing studies of different combinations of BRAF inhibitors with various immunotherapy agents such as high dose interleukin-2 or ipilimumab [48]. In November 2014 the study entitled “A Randomized Phase III Trial of Dabrafenib + Trametinib Followed by Ipilimumab + Nivolumab at Progression versus Ipilimumab + Nivolumab Followed by Dabrafenib + Trametinib at Progression in Patients With Advanced BRAF V600 Mutant Melanoma” was launched. Three hundred participants are expected to take part in the trial. The primary outcome of the trial is the OS rate, described as the proportion of patients alive after a 2-year follow-up. The completion date of the trial is estimated April 2016 [55].

### 3.2. MEK Inhibitors

#### 3.2.1. Trametinib

Trametinib, a highly selective MEK1/2 inhibitor, was approved by the FDA on May 29, 2013, as a first-line treatment for patients with unresectable or metastatic melanoma with V600E/K mutation. The approval was based on phase III multinational, randomized trial METRIC [56, 57]. The study measured the efficacy of trametinib in comparison to chemotherapy. The primary endpoint of the study was PFS in patients with BRAF V600E-mutant melanoma with no prior brain metastases. Secondary endpoints included ORR, OS, and safety profile of the drug. Overall, 322 patients with BRAF V600 E/K mutation were randomized into trametinib arm and chemotherapy (dacarbazine or paclitaxel) arm in a 2 : 1 manner. A total number of 273 patients were BRAF V600E positive with no history of brain metastases. The study showed noticeable improvement in PFS in the group of trametinib when compared with the chemotherapy group (4.8 months versus 1.4 months, resp.). The confirmed ORR was 24% for trametinib and 7% for chemotherapy. Hazard ratio (HR) for interim OS was 0.53 (95% CI, 0.30–0.94, *p* = 0.0181) in favor of trametinib group. The patients were allowed to cross over from chemotherapy group to trametinib group after confirmation of the disease progression (PD). The most commonly observed adverse events during the treatment included rash, diarrhea, edema, hypertension, and fatigue. The events typical of MEK inhibitors that could be noticed were chorioretinopathy (<1%) and the decrease of ejection fraction (7%). On the basis of the METRIC study, treatment with trametinib is associated with longer PFS when compared with chemotherapy (dacarbazine or paclitaxel) in patients with BRAF V600 E/K mutant melanoma [23].

#### 3.2.2. Selumetinib

Selumetinib, a highly selective MEK 1/2 inhibitor, has been tested in order to assess its efficacy and safety profile in numerous studies associated with various types of tumors. The combinations of selumetinib and different chemotherapeutic agents including irinotecan, docetaxel, temozolomide, and doxorubicin showed the enhanced activity against tumor cells in malignancies such as BRAF-mutant melanoma, non-small-cell lung cancer, pancreatic cancer, or hepatocellular carcinoma [58–61]. The study comparing selumetinib and temozolomide was carried out on patients with advanced mucosal or uveal melanoma, regardless of the status of BRAF mutations. Overall, 200 patients were enrolled in phase II of the study. They were randomized into selumetinib group, where they were administered the medicament in dose 100 mg twice daily and temozolomide group, where they were given temozolomide 200 mg/m^2^ daily for 5 days every 28 days [62]. The crossover from temozolomide to selumetinib was allowed in case of disease progression. The results of the study showed the comparable progression-free survival in both groups of 78 and 80 days for selumetinib and temozolomide, respectively (HR 1.07; 80% CI: 0.86–1.32). Moreover, there was no significant difference in PFS between 2 subgroups of BRAF- and/or NRAS-mutants. Partial responses were observed in 5.8% in selumetinib and 9.4% in temozolomide group. As for patients with BRAF mutations, objective responses did not vary noticeably between selumetinib and temozolomide arms (11.1% and 10.7%, resp.) [62]. Another phase II randomized trial was conducted to compare the effects of treatment with combinations of dacarbazine plus selumetinib and dacarbazine plus placebo. Overall, 91 previously untreated patients with advanced melanoma were enrolled. The crossover from one group to another in case of disease progression was not allowed during the study. Median OS was 13.9 months in the selumetinib plus dacarbazine group and 10.5 months in the placebo plus dacarbazine group (HR 0.93; 80% CI: 0.67–1.28; *p* = 0.39). The results of the study proved there was no improvement in survival after the addition of selumetinib to dacarbazine when compared to placebo and dacarbazine. In spite of the data mentioned above, PFS was noticeably longer in selumetinib plus dacarbazine group than in placebo plus dacarbazine group (5.6 months (80% CI: 4.9–5.9) versus 3.0 months (2.8–4.6), resp.) [63].

#### 3.2.3. Cobimetinib

Cobimetinib is a noncompetitive inhibitor, highly specific for MEK1 and 2 kinases. Phase III, randomized study has recently been conducted in order to compare the efficacy of the combination of vemurafenib and cobimetinib versus vemurafenib alone. The group of 495 previously untreated patients with BRAF V600-positive melanoma was randomized into vemurafenib plus cobimetinib and vemurafenib and placebo arms. The primary endpoint was investigator-assessed progression-free survival. The study showed the increased PFS in the combination group when compared to the control group with 9.9 months for cobimetinib plus vemurafenib versus 6.2 months for vemurafenib plus placebo. HR for death or disease progression was 0.51, 95% CI, 0.39–0.68; *p* < 0.001. The 9-month OS was also higher in the combination group than in the control group (81% versus 73%, resp.) [64]. Several other studies of vemurafenib and cobimetinib combination in various clinical conditions such as brain metastases, as an adjuvant therapy or with other agents like bevacizumab, are underway [65–69].

#### 3.2.4. MEK162

MEK162 is a highly selective MEK 1/2 kinases inhibitor. In a phase II study, conducted by Ascierto and coworkers, the activity against BRAF- and NRAS-mutant melanomas has been proved [70]. The number of 71 patients was enrolled and they were grouped into three cohorts depending on which mutation they harbored. Patients in each group were treated with MEK162: the NRAS-positive patients were administered 45 mg twice daily, while the BRAF-positive ones received either 45 or 60 mg twice daily. No analysis was possible in the 60 mg group as there were too few patients at data cutoff. As for the remaining cohorts, 30 and 41 patients with NRAS- and BRAF-mutant melanomas, respectively, received MEK162 45 mg twice daily. About 20% in both groups presented partial response. About 13% of NRAS-mutant patients and 27% of BRAF-mutant ones withdrew from the study as a result of the adverse events that occurred. The most commonly seen adverse events were acneiform dermatitis, rash, peripheral edema, facial edema, diarrhea, or elevated creatinine phosphokinase. About 18% of the patients developed serous retinopathy-like events. The substantial outcome of this study is that MEK162 is the first agent to be active against NRAS-mutant melanoma [70].

#### 3.2.5. Combined Therapy

As the mechanisms responsible for resistance to BRAF inhibitors have been discovered, numerous studies are being conducted so as to find methods of overcoming or delaying the resistance. Thus, the study measuring the efficacy of the combination of dabrafenib and trametinib in comparison with dabrafenib alone (COMBI-DT, phase III study) was conducted and as it showed the response rate of a single-agent treatment with dabrafenib was similar to the treatment with dabrafenib and trametinib in doses 150/1 mg daily but lower than the response rate of the treatment with dabrafenib and trametinib in doses 150/2 mg daily. However, the progression-free survival for dabrafenib alone was significantly lower when compared to the treatment with a combination of dabrafenib and trametinib in doses 150/1 mg daily and 150/2 mg daily (9.2 months for dabrafenib/trametinib 150/1 mg, 9.4 months for dabrafenib/trametinib 150/2 mg, and 5.8 months for dabrafenib 150 mg) [71]. On the basis of this trial, on January 2014, FDA approved the combination of trametinib and dabrafenib to treat patients with unresectable or metastatic melanoma with BRAF V600E or V600K mutation [72]. Another phase III trial (COMBI-V) comparing efficacy of the treatment with the combination of dabrafenib and trametinib to vemurafenib was conducted and the results have been published recently. In this study 704 participants were randomized 1 : 1 into dabrafenib plus trametinib arm and into vemurafenib arm, each consisting of 352 patients. The participants were administered either trametinib 2 mg once daily and dabrafenib 150 mg twice daily or vemurafenib 960 mg twice daily. In both arms the treatment continued until disease progression, death, unacceptable toxicities, or withdrawal of consent. Primary endpoint was OS and secondary endpoints included progression-free survival, overall response, and duration of response (all of them assessed by an investigator). The study revealed the prolonged overall survival for trametinib plus dabrafenib group as compared with the vemurafenib group being 18.3 and 17.2 months, respectively. PFS was also noticeably longer for patients in dabrafenib plus trametinib group than for those in vemurafenib group, 11.4 (95% CI, 9.9–14.9) versus 7.3 (95% CI, 5.8–7.8) months [24]. Moreover, numerous approaches testing combinations of BRAF/MEK inhibitors or BRAF/PI3K inhibitors are underway [48]. In October 2014, phase II, single-arm study testing the efficacy of the combination of dabrafenib and trametinib in patients previously treated with BRAF inhibitors was launched. The primary endpoint is the overall response rate, while the secondary endpoints are PFS, OS, and number of participants with adverse events. The study was planned for 25 patients and it is estimated to be completed by June 2016 [73]. The study of dabrafenib and vemurafenib used as an adjuvant therapy in patients with BRAF V600 mutation positive melanoma who underwent surgical treatment is also in progress. The study began in January 2013, and it is estimated to be completed in July 2015. Its primary endpoint is the relapse-free survival while secondary endpoints include overall survival, distant-metastasis free survival, and freedom from relapse. All these secondary endpoints are assigned to two cohorts, receiving either dabrafenib plus trametinib or placebo. Overall, 852 participants are expected to enroll [74]. Another study (COMBI-Neo) of combination of dabrafenib and trametinib in clinical stage III or oligometastatic (stage IV) melanoma is ongoing. The study is about to measure the efficacy of the mentioned combination given before the surgical treatment in comparison to having surgery alone. Overall, 84 patients are expected to take part in the study. The completion date is estimated October 2017 [75]. The other BRAF/MEK combinations that are currently underway are vemurafenib plus cobimetinib or LGX 818 plus MEK 162.

The results of selected clinical trials regarding MAPK-targeting agents are presented in (Table 1).

### 3.3. C-KIT Inhibitors

C-KIT is a receptor tyrosine kinase which activates the MAPK signaling pathways resulting in proliferative and survival effects. According to the study conducted by Curtin et al. [76] mutations of c-Kit are found in several types of melanoma, acral (36%), mucosal (39%), and sun-damaged (28%) melanoma. Phase II trial in patients with metastatic melanoma harboring c-KIT mutations or amplifications was conducted so as to test the efficacy of imatinib, a tyrosine kinase inhibitor. Overall, 43 patients participated in the trial. They were given imatinib in a dose of 400 mg/d until disease progression or unacceptable toxicities. The dose-escalation up to 800 mg/d was allowed in case of disease progression. The study resulted in a median PFS of 3.5 months while the 6-month PFS was 36.6%. A total of 53.5% of the patients achieved a response, 23.3% (*n* = 10) of whom achieved partial response (PR) and 30.2% (*n* = 13) of whom had stable disease (SD). In 41.9% of the participants regression of tumor mass could be noticed. The 1-year OS for the patients in this study was 51%. As it turned out, dose-escalation up to 800 mg/d presented no clinical benefit when compared with a dose of 400 mg/d [77].

There were also clinical trials testing the activity of other c-KIT inhibitors, such as sunitinib. This agent is a multitarget tyrosine kinase inhibitor. The investigators recruited 20 patients with metastatic uveal melanoma, expressing c-kit. Among them, there were 17 patients who failed previous treatment. Sunitinib was administered 37.5 mg daily in 4-week cycles. The results of the study showed that partial response was achieved by 1 patient (5%) but stable disease could be observed in 12 participants (60%) after sunitinib treatment. The median OS and PFS were 8.2 and 4.2 months, respectively. The conclusion of this study was that sunitinib may be a promising chance for patients with metastatic melanoma. The trials of other c-KIT inhibitors such as nilotinib and masatinib in melanoma are currently underway [78, 79].

## 4. Immunotherapy

### 4.1. CTLA-4 Antibodies

#### 4.1.1. Ipilimumab

Ipilimumab, a monoclonal antibody against CTLA-4, was approved by the FDA on March 25, 2011, to treat patients with unresectable or metastatic melanoma. The study that contributed most to the approval was a phase III randomized clinical trial comparing the efficacy of the combination of ipilimumab and glycoprotein 100 vaccine with ipilimumab or glycoprotein 100 alone [25]. Overall, 676 patients with HLA-A2^*∗*^0201 positive genotype participated in the study. They were randomized 3 : 1 : 1 into three arms, ipilimumab, 3 mg/kg intravenously, in combination with the tumor vaccine (*n* = 403), ipilimumab plus vaccine placebo (*n* = 137), and tumor vaccine with placebo (*n* = 136). All patients recruited to take part in the study had undergone systematic treatment for melanoma before the study. The overall survival was the trial's primary endpoint. The secondary endpoint included progression-free survival and overall response rate. The results of this trial show the clinical benefit in terms of the primary endpoint. The median OS for both groups of ipilimumab was 10.1 months (95% CI; 8.3–13.8) for ipilimumab alone and 10 months (95% CI; 8.5–11.5) for ipilimumab plus glycoprotein 100 vaccine while the median OS for glycoprotein 100 vaccine alone was significantly lower, 6.4 months (95% CI; 5.5–8.7). The best ORR was associated with the treatment with ipilimumab alone (10.9%; 95% CI, 6.3–17.4) versus 5.7% for ipilimumab plus glycoprotein 100 vaccine (95% CI, 3.7–8.4) versus 1.5% (95% CI; 0.2–5.2) for glycoprotein 100 vaccine alone [25, 80, 81]. In another phase III randomized study ipilimumab was compared to dacarbazine in terms of efficacy. A total number of 502 previously untreated patients with metastatic melanoma were enrolled and they were randomly assigned 1 : 1 into two arms of the study, receiving either ipilimumab (10 mg per kilogram) plus dacarbazine (850 mg per square meter of body-surface area) or dacarbazine (850 mg per square meter) plus placebo, given at weeks 1, 4, 7, and 10, followed by dacarbazine alone every 3 weeks through week 22. The primary endpoint of the study was overall survival. As the study results showed, the combination of dacarbazine plus ipilimumab was associated with a longer overall survival when compared with dacarbazine alone, 11.2 months versus 9.1 months, respectively, with higher survival rates in the ipilimumab-dacarbazine group at 1 year (47.3% versus 36.3%), 2 years (28.5% versus 17.9%), and 3 years (20.8% versus 12.2%) (hazard ratio for death, 0.72; *p* < 0.001) [26]. In a phase II study investigators compared the effect of ipilimumab plus sargramostim (GM-CSF) versus ipilimumab alone on overall survival in patients with advanced melanoma [27]. Overall, 245 patients with unresectable stage III or stage IV melanoma with the history of systemic treatment of the disease were recruited. The primary endpoint of this study was overall survival while the secondary endpoints included PFS, response rate, safety, and tolerability. As the results showed the overall survival was noticeably longer in the combination group than in ipilimumab group and it was 17.5 months (95% CI, 14.9 not reached) versus 12.7 months (95% CI, 10.0 not reached), respectively. In spite of that, the median PFS did not vary between the groups of the study being 3.1 months (95% CI, 2.9–4.6) versus 3.1 months (95% CI, 2.9–4.0) for ipilimumab plus GM-CSF and ipilimumab, respectively [27]. Ipilimumab was also tested as a drug in melanoma with brain metastases. A phase II study was conducted to assess the safety and efficacy of ipilimumab in patients with brain metastases. Overall, 72 patients were enrolled. They were assigned to two cohorts, neurologically asymptomatic with no ongoing treatment with steroids in cohort A (*n* = 51) and neurologically symptomatic on a stable dose of steroids in cohort B (*n* = 21). The primary endpoint of the study was the proportion of patients with disease control which consisted of partial response, complete response, or stable disease after 12 weeks. Among the patients in cohort A nine achieved disease control (18%, 95% CI; 8–31) and so did one patient in cohort B (5%; 0.1–24). When the brain alone was assessed, 12 patients in cohort A (24%, 13–38) and two in cohort B (10%, 1–30) achieved disease control [82]. The combinations of ipilimumab with various agents are subjects of numerous clinical trials. Phase II study of granulocyte macrophage-colony stimulating factor (GM-CSF) in combination with ipilimumab is currently ongoing. There are 43 patients expected to participate and the primary endpoint of the study is to assess the disease control rate at 24 weeks as defined by the immune-related response criteria (irRC) [83]. Phase I study of combination of bevacizumab and ipilimumab is currently underway. A number of 46 patients are about to take part in the study. The main objective of this trial is to determine safety of the treatment with both drugs together as well as the doses that can be administered to patients safely. The study was designed for patients with unresectable stage III or stage IV melanoma [84].

#### 4.1.2. Tremelimumab

Tremelimumab is a CTLA-4 blocking antibody. In phases I and II of the studies of tremelimumab there has been a promising response in patients under treatment [28]. The response rates were similar to ipilimumab and could be observed in about 10% of patients [28]. Notwithstanding the mentioned trials, phase III trial demonstrated no superiority over standard chemotherapy for advanced or metastatic melanoma in patients who underwent treatment with tremelimumab. Overall, 655 participants were recruited to take part in the trial. They were randomly assigned 1 : 1 to tremelimumab (15 mg/kg every 90 days) arm or chemotherapy arm where they were given dacarbazine or temozolomide in standard doses. The median OS for tremelimumab arm was 12.6 months (95% CI; 10.8–14.3) versus 10.7 months for chemotherapy arm (95% CI; 9.36–11.96). The response rates for both groups were even more similar being 10.7% and 9.8% for tremelimumab and chemotherapy, respectively. Despite the insignificant differences between the depicted measurements, the duration of response was noticeably longer for tremelimumab than for chemotherapy (35.8 versus 13.7 months, resp.). The study did not present survival advantage for tremelimumab when compared with standard chemotherapy [29] which most probably resulted in lack of FDA approval of this agent.

### 4.2. PD-1 Inhibitors

#### 4.2.1. Nivolumab

Nivolumab is a fully human monoclonal antibody against PD-1 (programmed death receptor-1). It has been approved by FDA recently (December 22nd 2014) to treat unresectable or metastatic melanoma with no response to other drugs [85]. A noncomparative phase II study was conducted in Japan assessing the efficacy of nivolumab. In this study almost 25% of participants, who had stages III/IV (unresectable or metastatic) melanoma, received partial tumor response while being treated with nivolumab. The median PFS in the study was 172 days [30]. The results of phase III study comparing nivolumab to standard chemotherapy with dacarbazine have recently been published [31]. Overall, 418 previously untreated patients with BRAF-positive melanoma took part in the study. They were randomized into two groups, nivolumab group (receiving nivolumab at a dose of 3 mg per kilogram of body weight every 2 weeks and dacarbazine-matched placebo every 3 weeks) and dacarbazine group (receiving dacarbazine at a dose of 1000 mg per square meter of body-surface area every 3 weeks and nivolumab-matched placebo every 2 weeks). OS was the primary endpoint of the study. At 1 year the median OS was 72.9% for nivolumab group (95% CI; 65.5–78.9) and 42.1% for dacarbazine group (95% CI; 33.0–50.9). Hazard ratio for death was 0.42; 99.79% CI, 0.25 to 0.73; *p* < 0.001. The response rate was also noticeably higher for nivolumab than for dacarbazine, 40.0% (95% CI, 33.3 to 47.0) versus 13.9% (95% CI, 9.5 to 19.4), respectively. Not only did nivolumab present the superior clinical effect over dacarbazine, but also it had a better safety profile. Adverse events associated with the treatment occurred in 11.7% of the patients in nivolumab group and 17.6% of the patients in dacarbazine group [31]. Another study testing the combination of nivolumab and ipilimumab was conducted [86]. Patients in this study received either nivolumab and ipilimumab every 3 weeks for 4 doses, followed by nivolumab alone every 3 weeks for 4 doses (concurrent regimen) or nivolumab every 2 weeks for up to 48 doses if they had been treated with ipilimumab before (sequenced regimen). A total number of 86 patients were enrolled, 53 in concurrent regimen and 33 in sequenced regimen. The objective response rate for concurrent-regimen group was 40%. Evidence of clinical activity described as conventional, unconfirmed, or immune-related response or stable disease for at least 24 weeks was observed in 65% of patients in the concurrent-regimen group. In comparison, the ORR in sequenced-regimen group was only 20%. To sum up, the concurrent therapy with ipilimumab and nivolumab is associated with higher response rate when compared to monotherapy [86]. Thus, phase III study of combination of nivolumab and ipilimumab in comparison with either nivolumab or ipilimumab alone has been launched. Overall, 915 previously untreated patients with unresectable stage III or stage IV melanoma are expected to participate in the study. The completion date is estimated September 2016 [87]. The trial of nivolumab and ipilimumab in combination with sargramostim is about to start. The investigators expect 400 patients with unresectable stage III or IV melanoma to be enrolled. They are supposed to be randomized into ipilimumab and nivolumab plus sargramostim (GM-CSF) group and ipilimumab plus nivolumab group. The primary endpoint of the study is OS. The completion date is estimated March 2016 [88].

#### 4.2.2. Pembrolizumab/Lambrolizumab (MK-3475)

Pembrolizumab (formerly named lambrolizumab or MK-3475) is a highly selective humanized IgG4-*κ* isotype monoclonal antibody against PD-1. Its efficacy in advanced melanoma has been proved in a phase IB study, where 135 patients divided into three cohorts were administered 10 mg/kg every 2 weeks, 10 mg/kg every 3 weeks, or 2 mg/kg every 3 weeks. The ORR was measured according to RECIST. The study showed that ORR for all three cohorts was 38%, but the highest response rate was associated with the dose of 10 mg/kg every 2 weeks and it was 52%. The median PFS was over 7 months. The response rate in the group of patients with no prior exposure to ipilimumab therapy was similar to the one observed in those who had undergone previous treatment with ipilimumab and it was 37% and 38%, respectively [32, 33]. Another phase I trial of pembrolizumab in patients with refractory melanoma was conducted. In this study 173 patients were randomized to receive either 2 mg/kg or 10 mg/kg every three weeks. As the study shows, the median response rate for both groups was 26% (difference 0%, 95% CI −14 to 13; *p* = 0.96). The median PFS was 22 weeks (95% CI 12–36) for the pembrolizumab 2 mg/kg group and 14 weeks (12–24) for the pembrolizumab 10 mg/kg group (HR 0.84, 95% CI 0.57–1.23). The results of the study mentioned above may be considered as very promising for patients who did not respond to treatment with ipilimumab administered as a first-line therapy [34]. Several other studies regarding pembrolizumab are now underway testing the efficacy of pembrolizumab in advanced melanoma [89] or comparing the agent with chemotherapeutic agents such as carboplatin, pacliaxel, dacarbazine, or temozolomide [90]. On September 4, 2014, the US FDA approved pembrolizumab for the treatment of patients with unresectable or metastatic melanoma with disease progression after treatment with ipilimumab or BRAF-inhibitor in case of BRAF-positive disease [91].

#### 4.2.3. Pidilizumab

Pidilizumab (CT-011), another PD-1 inhibitor, is a humanized monoclonal IgG1-*κ* antibody. Its activity in patients with metastatic melanoma was measured in a phase II randomized study and the results were reported in 2014. The total number of 103 patients participated in the trial. They were assigned to two dose-level groups, 1.5 and 6 mg/kg administered every two weeks for 27 weeks. The results showed that the ORR for patients treated with pidilizumab was only 6%, which was lower than the ones observed in studies regarding the other PD-1 inhibitors. The 12-month OS was 65% with no significant difference between the doses tested as well as between the ipilimumab-naive or ipilimumab-treated patients [35].

### 4.3. PD-L1 Inhibitors

#### 4.3.1. MPDL3280A

MPDL3280A, a human monoclonal antibody against PD-L1, blocks the binding of PD-L1 to PD-1 and B7-protein. Its efficacy has been tested in metastatic melanoma and there are also numerous trials underway. A study on 45 patients who were administered doses ranging from 1 to 20 mg/kg was conducted [92]. The ORR measured in this study was observed in 26% of the patients and the 24-week PFS was about 35%. The treatment with MPDL3280A was well tolerated and, what is more significant, associated with durable responses [92]. Currently, there are several studies in progress associated with MPDL3280A in melanoma [93, 94]. One of them, a phase Ib study of combination of MPDL3280A and vemurafenib in comparison with vemurafenib and cobimetinib is recruiting participants. The primary outcome of the trial is to assess dose-limiting toxicities and analyze the adverse effects regarding the treatment. The study is about to be completed in 2017 [69].

#### 4.3.2. BMS-936559

BMS-9 36559 is a fully human IgG4 PD-L1 antibody. Its clinical activity was tested in a phase I study on 207 patients and the efficacy of the drug in melanoma was assessed in 52 patients. The patients were treated with different doses of BMS-936559 ranging from 0.3 mg/kg to 10 mg/kg. The response rates varied depending on the dose the patient was given. The response rates were 6% for 1 mg/kg, 29% for 3 mg/kg, and 19% for 10 mg/kg. Nine patients from melanoma group achieved a response and three of them achieved a complete response. Five out of nine patients mentioned above had an object response for over a year and 27% (14 out of 52) of all patients with melanoma participating in this study had stable disease for at least 24 weeks [95].

#### 4.3.3. MEDI4736

MEDI4736, another humanized IgG-1*κ* monoclonal antibody which blocks PD-L1, is currently a subject of clinical trials [96, 97]. The interim results of an ongoing phase I study conducted to assess safety, tolerability, and pharmacokinetics of this agent have recently been reported. Among the 26 participants receiving dose-escalation treatment with MEDI4736 no dose-limiting toxicities were observed. A total of 34% of participants developed treatment-related adverse events, none of which led to treatment discontinuation. Partial response could be seen in 4 of the 26 patients. The ORR (partial response and stable disease for at least 12 weeks) was 46% [96]. Another phase I study testing the combination of MEDI4736 and tremelimumab is currently underway. A total number of 102 patients are supposed to enroll. The study is designed to measure safety and tolerability of the mentioned combination in advanced solid tumors. The primary outcome of the study is a number of adverse events. The data completion date is estimated December 2016 [97].

#### 4.3.4. MSB0010718C

MSB0010718C is a fully humanized IgG1 monoclonal antibody blocking PD-L1. The early phase clinical studies are currently underway, testing the safety, tolerability, and pharmacokinetics of this agent in patients with advanced solid tumors. The number of 27 patients were recruited and they took part in a dose-escalation study. The follow-up for at least 4 weeks was possible for 23 patients. Discontinuation of the treatment occurred in 12 patients (52.2%), in 9 of whom due to disease progression, 2 (8.7%) because of adverse events, and 1 (4.3%) because of the patient's death. The adverse events of grade 3 or 4 were observed in 3 patients. One patient developed dose-limiting toxicities (DLT) at dose 20 mg/kg [98].

#### 4.3.5. AMP-224

AMP-224 is a recombinant B7-DC-Fc fusion protein that modulates the PD-1 axis by depleting PD-1 high expressing effector T-cells. This mechanism is thought to restore the immune function to T-cells. Its action was assessed in a phase I study, where 42 patients were treated with various doses of AMP-224 ranging from 0.3 mg/kg to 30 mg/kg [99, 100]. The primary outcome of this study included a number of patients with adverse events and a number of participants with dose-limiting toxicities or laboratory changes, as well as it was meant to determine the maximum tolerated dose and the recommended phase II dose [100]. The study has been completed and the results are pending.

The outcomes of the clinical studies of immunotherapeutic agents mentioned in the text are shown in (Table 2).

## 5. Conclusion

Poor prognosis for patients with melanoma has changed radically over past few years. The approaches in use are becoming more and more promising as several novel agents designed for patients with advanced melanoma appeared just a few years ago. Numerous clinical studies present clinical benefit of treatment with new medicaments over standard chemotherapeutics. Despite the spectacular success of the biological agents designed for treatment of melanoma, a problem of resistance to treatment is a major challenge for clinicians. Thus, the results of numerous trials of various combinations of recently approved drugs or new promising agents are yet to be published. That means patients diagnosed with melanoma are given a chance to get sophisticated treatment that has never been accessible before.

## Figures and Tables

**Table 1:** Summary of selected clinical trials of MAPK-targeting agents.

Phase	Drug Name	Study design	Patient population	No. pts	ORR (%)	PFS (months)	OS (months)	Reference
I	Vemurafenib	Vemurafenib dose-expansion (BRIM-1)	Metastatic melanoma with BRAF V600E mutation	32	81	7	13.8	[16]

II	Vemurafenib	Vemurafenib (BRIM-2)	Metastatic melanoma with BRAF V600 mutation	132	53	6.8	15.9	[17]

III	Vemurafenib	Vemurafenib vs Dacarbazine (BRIM-3)	Previously untreated patients with BRAF V600-mutant positive melanoma (stage IIIc/IV)	675	48 vs 5	5.3 vs 1.6	13.2 vs 9.6	[18, 19]

II	Dabrafenib	Dabrafenib in V600E- vs V600K-mutant pts (BREAK-2)	V600E- and V600K-mutant melanoma	92	59 vs 13	6.3 vs 4.5	13.1 vs 12.9	[20]

III	Dabrafenib	Dabrafenib vs Dacarbazine (BREAK-3)	Unresectable stage III or IV melanoma with BRAF V600E-mutation	250	50 vs 6	5.1 vs 2.7	NA	[21, 22]

III	Trametinib	Trametinib vs Dacarbazine	Stage IIIc or IV melanoma with BRAF V600E/K mutation	322	24 vs 7	4.8 vs 1.4	NA	[23]

III	Dabrafenib, Vemurafenib, Trametinib	Dabrafenib + Trametinib vs Vemurafenib	Unresectable or metastatic cutaneous melanoma with BRAF V600E/K mutation	704	64 vs 51	11.4 vs 7.3	18.3 vs 17.2	[24]

**Table 2:** Summary of selected clinical trials of immunotherapeutic agents for melanoma treatment.

Phase	Drug Name	Study design	Patient population	No. pts	ORR (%)	PFS (months)	OS (months)	Reference
III	Ipilimumab	Ipilimumab vs Ipilimumab plus GP100 vs GP100	Previously treated advanced melanoma	676	10.9 vs 5.7 vs 1.5	2.86 vs 2.76 vs 2.76	10.1 vs 10.0 vs 6.4	[25]

III	Ipilimumab, Dacarbazine	Ipilimumab plus dacarbazine vs dacarbazine	Previously untreated metastatic melanoma	502	15.2 vs 10.3	NA	11.2 vs 9.1	[26]

II	Ipilimumab, GM-CSF	Ipilimumab plus GM-CSF vs ipilimumab	Metastatic melanoma	245	NA	3.1 vs 3.1	17.5 vs 12.7	[27]

I/II	Tremelimumab	Dose-escalation of tremelimumab	Unresectable stage III or stage IV melanoma	117	9.8 (10 mg/kg)	NA	9.97 (10 mg/kg), 11.53 (15 mg/kg)	[28]

III	Tremelimumab	Tremelimumab vs dacarbazine/temozolomide	Previously untreated stage IIIc or IV melanoma	655	10.7 vs 9.8	Duration of response: 35.8 vs 13.7	12.6 vs 10.7	[29]

II	Nivolumab	Nivolumab	Unresectable stage III or IV melanoma	90	25	172 days	Not yet reached	[30]

III	Nivolumab, Dacarbazine	Nivolumab vs Dacarbazine	Previously untreated BRAF-mutation positive melanoma	418	40.0 vs 13.9	5.1 vs 2.2	At 1-year 72.9% vs 42.1%	[31]

I	Pembrolizumab	Safety/efficacy of three doses	Advanced melanoma	135	38/37 (by IRC)	Over 7 months	Not reached	[32, 33]

I	Pembrolizumab	Pembrolizumb 2 mg/kg/10 mg/kg	Refractory melanoma	173	26	22/14 weeks	NA	[34]

II	Pidilizumab	Pidilizumab 1.5 mg/kg/6 mg/kg	Metastatic melanoma	103	6	2.8/1.9	1 year: 65%	[35]
